# Arsenic Species Analysis at Trace Level by High Performance Liquid Chromatography with Inductively Coupled Plasma Mass Spectrometry

**DOI:** 10.1155/2019/3280840

**Published:** 2019-06-04

**Authors:** Hongfang Hou, Wanjing Cui, Qing Xu, Zhanhui Tao, Yafei Guo, Tianlong Deng

**Affiliations:** Tianjin Key Laboratory of Marine Resources and Chemistry, Modern Analytical Technology Research Center, College of Chemical Engineering and Materials Science, Tianjin University of Science and Technology, Tianjin 300457, China

## Abstract

A sensitive and accurate simultaneous continuous analysis for six arsenic species including arsenobetaine (AsB), arsenocholine (AsC), monomethylarsonic acid (MMA), dimethylarsinic acid (DMA), arsenite (As^III^), and arsenate (As^V^) has been developed by high performance liquid chromatography with inductively coupled plasma mass spectrometry (HPLC-ICP-MS). An anion-exchange column of Hamilton PRP-X100 (Switzerland) was applied for separation of the six arsenic species with gradient elution of 1.25 mmol/L Na_2_HPO_4_ and 11.0 mmol/L KH_2_PO_4_ as the mobile phase A and 2.5 mmol/L Na_2_HPO_4_ and 22.0 mmol/L KH_2_PO_4_ as the mobile phase B. The linearity ranges for AsB, AsC, MMA, DMA, As^III^, and As^V^ were between 0.5 and 50.0 *μ*g/L, and the detection limits of the six arsenic species were all within 0.01–0.35 ng/L. The relative standard deviations (RSDs) were within 2.26–3.68% and the recovery rates of samples ranged from 95 to 103%. The proposed method was applied for the arsenic speciation analysis of sediment pore-water samples, which were taken from the supernatant after centrifugation and filtration.

## 1. Introduction

Arsenic as a typical toxic element [1, 2] is considered as one of the primary pollutants, and the detriment of arsenic to human body is secular and chronic. Mcneill and Edwards [3] reported that toxicity of arsenite (As^III^) is 60 times higher than that of arsenate (As^V^), and the organic forms such as dimethylarsinic acid (DMA) and monomethylarsonic acid (MMA) are much less toxic [4] and the sequence of toxicity is in order of AsH_3_ > As^III^ > As^V^ > MMA > DMA > AsB ≈ AsC [5]. In natural environment, arsenic in sediment, water body, and atmosphere mainly existed in the form of inorganic compounds including arsenate and arsenite.

As we well know, most of speciation analytical methods in the literature are based on separation techniques hyphenated with high-precision detectors, for instance, capillary electrophoresis (CE) [6], ion chromatography (IC) [7], or high performance liquid chromatography (HPLC) [8], coupled to atomic fluorescence spectrometry (AFS) [9], atomic absorption spectrometry (AAS) [10], or inductively coupled plasma mass spectrometry (ICP-MS) [11]. Nevertheless, simultaneous continuous measurement of six arsenic species is still crucial but challenging. Compared with other combined technologies, high performance liquid chromatography (HPLC) hyphenated with ICP-MS is an ultrasensitive method for the determination of all arsenic species in complex sediments [12–14] since ICP-MS has an advantage that it can achieve trace analysis. It is worth noting that ICP-MS has been proverbially used in all kinds of samples such as in urine [15, 16], ground water [17, 18], and food [19, 20], but the arsenic species simultaneous continuous measurement using HPLC-ICP-MS in the sediment pore-water samples has not been reported. In this work, we present a practical and sensitive method for the quantification of arsenite, arsenate, and organic arsenic species in sediment pore-water samples.

## 2. Experimental

### 2.1. Apparatus

An iCAP Q ICP-MS (Thermo Scientific, USA) was used for quantitative analysis of arsenic speciation. A Thermo U3000 HPLC system was successfully used to separate arsenic species with the injection volume of 20 *μ*L in this study. Separation of arsenic species was achieved using a Hamilton PRP-X100 column (250 mm length × 4.1 mm i.d, 10 *μ*m particle size) and the column temperature was at room temperature. A buffer solution of sodium hydrogen phosphate and monopotassium phosphate was used through gradient elution for separation of arsenic of six species. The outlet of the chromatographic column was forthright connected to the concentric nebulizer using a 0.18 mm i.d. PEEK tubing. ICP-MS was fitted with Kinetic Energy Discrimination (KED) mode allowing target isotope ions to enter the mass analyzer while preventing polyatomic interfering ions from entering the mass analyzer. By this way, background values can be reduced and obtain good peak shape. The HPLC and ICP-MS system working conditions were summarized in Table 1. The pH value was measured by a high-precision pH meter (WTW PH-7310, Shanghai Precision Scientific Instruments Co. Ltd., China) with an uncertainty of ± 0.003.

### 2.2. Reagents and Standard Solution

Ultrapure water (resistivity, 18.2 MΩ cm^−1^) obtained from a Milli-Q ultrapure water purification system (Millipore, Bedford, MA, USA) was used for all dilution in the experiment. Sodium hydrogen phosphate (A.R., Sinopharm Chemical Reagent Co., Ltd) and monopotassium phosphate (A.R., Sinopharm Chemical Reagent Co., Ltd) were used to prepare mobile phase, which were filtered through a 0.45 *μ*m membrane filter and bubbles were excluded in an ultrasonic bath before use. Standard solutions of AsB, AsC, MMA, DMA, As^III^, and As^V^ were purchased from Chinese Academy of Metrology (Beijing). Six species of arsenic stock solution (1.00 mg/L) were prepared and stored in polytetrafluoroethylene bottle at 4°C in the refrigerator. Working standard solution of 0–50 *μ*g/L was prepared from stock solution by gradient dilution for calibration curve prior to use and stored at 4°C.

### 2.3. Sample Collection and Preparation

Teflon fiber membrane balance sampler was adopted to collect samples, which was described in detail [22]. The sampler was immersed with 1% nitric acid for 3 days, then washed with distilled water, packed in plastic bags, and tightly wrapped in preservative bags in the laboratory. There was one sediment sample chosen in Tuojiang River in the west of China (104°31′19.0′′E, 30°43′44.2′′N). The core sampler was inserted into sediment slowly to sample about 20 to 25 cm in length and then carefully placed into the big plastic bag filled with nitrogen gas in situ, and then the samples were divided centimeter by centimeter from the bottom to the top with plastic knife to load into a series of numbering high-density polyethylene bottles and stored in low temperature preservation to move to laboratory quickly. For analysis, the samples were thawed firstly in the glove box (UNIlab Plus, MBraun, Germany) with nitrogen gas filled and refrigerated centrifuge (HERMLE Z326K, Germany) at speed of 18000 rpm, the pore-water sample was filtered with 0.45 *μ*m membrane into the high-density polyethylene bottle, and then a certain amount of hydrochloric acid was added to make the pH about 2 and then stored at 4°C for analysis as quickly as possible.

### 2.4. Speciation Analysis Procedures for Water Samples

For the speciation analysis of sediment pore-water samples collected in the high-density polyethylene bottles, the samples were removed from the refrigerator and returned to the room temperature without any other treatment and then directly injected using manual injection needle with quantitative loop volume of 20 *μ*L. The concentrations of six arsenic species in the pore-water sample were determined directly by HPLC-ICP-MS.

## 3. Results and Discussion

### 3.1. Selection of Chromatographic Conditions

**Figure 1:**
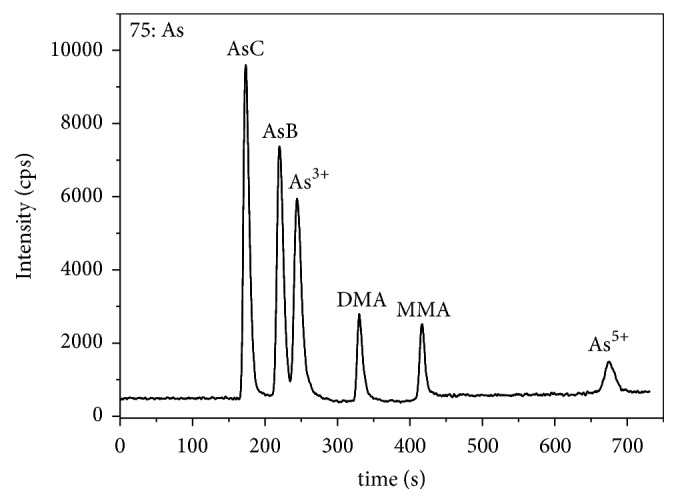
Chromatograms of arsenic speciation by gradient elution using mobile phase A: 1.25 mmol/L of Na_2_HPO_4_ and 11 mmol/L of KH_2_PO_4_; mobile phase B: 2.5 mmol/L of Na_2_HPO_4_ and 22 mmol/L of KH_2_PO_4_, 20 *μ*g/L for each arsenic speciation.

The chromatographic column and buffer solution as mobile phase were indispensable to establish a successful separation and analysis method for arsenic species using high performance liquid chromatography with inductively coupled plasma mass spectrometry (HPLC-ICP-MS). For the anion-exchange column, the retention of arsenic species can be influenced by the type of column, the ionic strength, concentration, and the flow rate of the mobile phase. In order to find the optimal mobile phase, Na_2_HPO_4_ (5 mmol/L) and KH_2_PO_4_ (44 mmol/L) were used to achieve a preferable separation of the arsenic species. AsC, MMA, DMA, and As^V^ were completely separated and the analysis time was abbreviated, but it was worth noting that AsB and As^III^ were not fully separated from each other. Meanwhile considering that the sampler and skimmer cones of ICP-MS would be blocked if the sodium salt concentration was too high, resulting in signal suppression [23], reducing the mobile phase concentration was adopted to analyze the arsenic species. Fortunately, AsB and As^III^ were fully separated; at the same time the analysis time would be applicable. Therefore, a gradient elution procedure consisting of 1.25 mmol/L of Na_2_HPO_4_ and 11 mmol/L of KH_2_PO_4_ and 2.5 mmol/L of Na_2_HPO_4_ and 22 mmol/L of KH_2_PO_4_ was adopted for the optimized chromatographic operating conditions in this experiment, and the procedure was listed in Table 1. The separation result is shown in Figure 1.

The Hamilton PRP-X100 anion-exchange chromatographic column was based on polymer anion-exchange filler, in which the elution behavior can be explicated by means of ion exchange mechanism. In order to discuss the elution behavior as described in this paper, the ionic forms of arsenic were estimated using the dissociation constants and the p*Ka* values of each species were shown in Table 2. Each speciation of arsenic has different anion-exchange capacity due to different p*Ka* value; the smaller the value is, the easier the corresponding acids dissociate and the stronger the retention in the column is. AsC, a cation irrespective of the pH, implied that the elution was almost unaffected; hence, it was the first form to be separated. Since AsB existed as the zwitterionic form while As^III^ existed as a weakly ionized compound [21], the interaction with stationary phase was weak, so AsB and As^III^ cannot be completely separated through high concentration in the mobile phase. Owing to the fact that other three arsenic species have low p*Ka* values in descending order of DMA, MMA, and As^V^, they sequentially appeared at the end in the separation process.

### 3.2. Analytical Performances

Under the optimum conditions, six arsenic species were achieved with the symmetrical peaks and have a good resolution. The analytical performance using the HPLC-ICP-MS was determined by the linearity of calibration curves, detection limits, and relative standard deviation. The calibration plot was obtained by drawing peak area of signal (cps) against the concentration of the homologous target ions. In Table 3, it was shown that the linearity of six arsenic species ranged from 0.5 to 50.0 *μ*g/L with 20 *μ*L of standard solution injection.

The calibration curve has achieved good linearity with correlation coefficients (*r*) values more than 0.9999. Method detection limits (3*σ*/*k*) were calculated, where* σ *was the standard deviation for three replicates of the lowest concentration of standard solution and* k* was slope of the calibration plot, ranging from 0.01 to 0.35 ng/L. The detection limit can be increased with a larger volume injection. Nevertheless, a larger volume injection can bring about column overloading and make the salt accumulation on the skimmer and sampler cones, which caused sensitivity reduction. The reproducibility was expressed by calculating the RSD of five repeated experiments using 20 *μ*g/L of standard mixture solution of each arsenic species. The results showed that RSDs for AsB, AsC, As^III^, DMA, MMA, and As^V^ were 3.68, 3.04, 3.19, 3.02, 3.36, and 2.26%, respectively. The results demonstrated that satisfactory reproducibility and sensitivity were achieved for arsenic speciation using this method.

### 3.3. Analytical Application

According to the speciation analysis procedures in Section 2.4, the proposed method was employed to determine arsenic speciation and corresponding content in sediment pore-water samples in depths of 0, -5, and -10 cm (from the surface to the depth of bottom) in Tuojiang River.

The analytical results of arsenic species and the recoveries of different species in the sediment pore-water samples via standard adding are shown in Table 4. The analytical results indicated that the concentrations of AsB and AsC were not detected in the whole core samples. And the three replicates of samples and standard additions through biking in the samples showed that recoveries for the six arsenic species of AsB, AsC, As^III^, DMA, MMA, and As^V^ were between 95 and 103%, and the RSDs for parallel experiments were below 3%. Furthermore, with the increase of underground depths, the concentration of As^III^ and MMA increased, the concentration of As^V^ decreased, and that of DMA changed indistinctively. Those results indicated that the proposed method in this work is valid for the determination of arsenic species in water samples.

## 4. Conclusions

In this work, it was clearly shown that high performance liquid chromatography with inductively coupled plasma mass spectrometry (HPLC-ICP-MS) can be perfectly applied in the determination of six arsenic species (AsB, AsC, MMA, DMA, As^III^, and As^V^) by one single anion- exchange column using sodium hydrogen phosphate and monopotassium phosphate as eluent. In order to avoid clogging sampler and skimmer cones, arsenic species were separated by reducing mobile phase concentration and setting gradient elution procedure for further optimization. In addition, operating mode of ICP-MS was adapted with Kinetic Energy Discrimination (KED) to improve instrument sensitivity. Under the optimized conditions, determination of six arsenic species has been achieved with good repeatability, high precision, and low detection limits. The proposed method was sufficient to detect the arsenic species and determine the corresponding concentration of each species in sediment pore water, whose form mainly existed in MMA, DMA, As^III^, and As^V^, and this method has also been validated accurately by recovery tests in sediment pore-water samples.

## Figures and Tables

**Table 1:** Working conditions of the HPLC and ICP–MS system.

Parameters	Value
ICP-MS system	
RF power (W)	1500
Cool flow (L min^−1^)	14.00
Auxiliary gas flow (L min^−1^)	0.80
Nebuliser gas flow (L min^−1^)	1.015
Collision gas flow (L min^−1^)	He/4.09
CCT1 flow (mL min^−1^)	5.198
Sampler depth (mm)	5.0
Spray chamber temperature/°C	2.70
Operation mode	KED mode
Isotopes monitored	^75^As
Dwell time (ms)	200
HPLC system	
Column	Hamilton PRP–X100 column (250 mm × 4.1 mm i.d., 10 *μ*m)
Mobile phase	Phase A: 1.25 mmol/L of Na_2_HPO_4_ and 11 mmol/L of KH_2_PO_4_, pH 6.116
	Phase B: 2.5 mmol/L of Na_2_HPO_4_ and 22 mmol/L of KH_2_PO_4_, pH 6.057
Flow–rate (mL/min)	Gradient elution of range 0.6-1.5 mL/min
HPLC elution program	0-4.9 min:100% A, 4.9-5.3 min: 100% A to 100% B, 5.3-12 min:100% B 0-4.3 min: 0.6 mL/min, 4.3-4.6 min: 0.6 to 1.0 mL/min, 4.6-4.9 min: 1.0 to 1.5 mL/min, 4.9-12 min: 1.5 mL/min.
Column temperature (°C)	Room temperature
Quantitative loop (*μ*L)	20 *μ*L

**Table 2:** p*Ka* values and formulas of arsenic species [21].

Species	Formula	p*Ka*
AsC	(CH_3_)_3_As^+^CH_2_CH_2_OH	−
AsB	(CH_3_)_3_As^+^CH_2_COOH	2.18
As^III^	H_3_AsO_3_	9.28
DMA	(CH_3_)_2_AsO(OH)_2_	6.3
MMA	CH_3_AsO(OH)_2_	2.6, 8.2
As^V^	H_3_AsO_4_	2.3, 6.8, 11.6

**Table 3:** Analytical figures of arsenic speciation by HPLC–ICP–MS.

Parameters	Analytical features					
	AsB	AsC	As^III^	DMA	MMA	As^V^
Linear range (*µ*g/L)	0.5–50.0	0.5–50.0	0.5–50.0	0.5–50.0	0.5–50.0	0.5–50.0
Coefficient (*r*)	1.0000	0.9999	1.0000	1.0000	1.0000	1.0000
LOD (ng/L)	0.01	0.05	0.11	0.28	0.20	0.35
R.S.D. (%, *n *= 5)	3.68	3.04	3.19	3.02	3.36	2.26

**Table 4:** Analytical results and recoveries of the sediment pore-water samples.

Depth (cm)	Arsenic species	Concentration (*µ*g/L) ± S.D.^a^	Added (*µ*g/L)	Found (*µ*g/L)	Recovery (%)
0	AsB	N.D.^b^	0.500	0.503 ± 0.002	100.6
	AsC	N.D.^b^	0.500	0.495 ± 0.005	99.0
	As^III^	0.217 ± 0.003	0.500	0.708 ± 0.002	98.2
	DMA	0.530 ± 0.002	0.500	1.032 ± 0.009	100.4
	MMA	0.012 ± 0.002	0.100	0.115 ± 0.007	103.0
	As^V^	2.431 ± 0.020	2.500	4.970 ± 0.008	101.6
-5	AsB	N.D.^b^	0.500	0.499 ± 0.006	99.8
	AsC	N.D.^b^	0.500	0.501 ± 0.003	100.2
	As^III^	0.389 ± 0.014	0.500	0.872 ± 0.011	96.6
	DMA	0.563 ± 0.005	0.500	1.061 ± 0.021	99.6
	MMA	0.019 ± 0.004	0.100	0.114 ± 0.006	95.0
	As^V^	2.302 ± 0.018	2.500	4.862 ± 0.029	102.4
-10	AsB	N.D.^b^	0.500	0.489 ± 0.009	97.8
	AsC	N.D.^b^	0.500	0.497 ± 0.005	99.4
	As^III^	0.447 ± 0.006	0.500	0.952 ± 0.010	101.0
	DMA	0.534 ± 0.010	0.500	1.040 ± 0.004	101.2
	MMA	0.074 ± 0.009	0.100	0.169 ± 0.013	95.0
	As^V^	2.057 ± 0.015	2.500	4.562 ± 0.004	100.2

^a^The values are presented as average ± confidence interval (*n* = 3).

^b^Not detectable.

## Data Availability

The data used to support the findings of this study are included within the article.
